# An NMPC-Based Integrated Longitudinal and Lateral Vehicle Stability Control Based on the Double-Layer Torque Distribution

**DOI:** 10.3390/s24134137

**Published:** 2024-06-26

**Authors:** Xu Bai, Yinhang Wang, Mingchen Jia, Xinchen Tan, Liqing Zhou, Liang Chu, Di Zhao

**Affiliations:** College of Automotive Engineering, Jilin University, Changchun 130025, Chinachuliang@jlu.edu.cn (L.C.);

**Keywords:** commercial vehicles, double-layer torque distribution, integrated control strategy, vehicle stability control

## Abstract

With the ongoing promotion and adoption of electric vehicles, intelligent and connected technologies have been continuously advancing. Electrical control systems implemented in electric vehicles have emerged as a critical research direction. Various drive-by-wire chassis systems, including drive-by-wire driving and braking systems and steer-by-wire systems, are extensively employed in vehicles. Concurrently, unavoidable issues such as conflicting control system objectives and execution system interference emerge, positioning integrated chassis control as an effective solution to these challenges. This paper proposes a model predictive control-based longitudinal dynamics integrated chassis control system for pure electric commercial vehicles equipped with electro–mechanical brake (EMB) systems, centralized drive, and distributed braking. This system integrates acceleration slip regulation (ASR), a braking force distribution system, an anti-lock braking system (ABS), and a direct yaw moment control system (DYC). This paper first analyzes and models the key components of the vehicle. Then, based on model predictive control (MPC), it develops a controller model for integrated stability with double-layer torque distribution. The required driving and braking torque for each wheel are calculated according to the actual and desired motion states of the vehicle and applied to the corresponding actuators. Finally, the effectiveness of this strategy is verified through simulation results from Matlab/Simulink. The simulation shows that the braking deceleration of the braking condition is increased by 32% on average, and the braking distance is reduced by 15%. The driving condition can enter the smooth driving faster, and the time is reduced by 1.5 s~5 s. The lateral stability parameters are also very much improved compared with the uncontrolled vehicles.

## 1. Introduction

With the rapid development of the automotive industry, electric vehicles are gradually becoming ideal platforms for the application and promotion of new technologies [1,2,3]. With the advancement of vehicle intelligence and the progress of electrification, various active safety features and advanced actuators are being developed. At the same time, the architecture of vehicle control strategies is also facing new challenges. In traditional distributed control architectures, the increase in vehicle motion control functions and hardware upgrades have led to higher system complexity, as well as resulting interference of control objectives and coupling of control inputs [4,5,6]. Therefore, it is crucial to develop integrated chassis control strategies that can eliminate subsystem conflicts and compensate for the limitations of each subsystem.

The main current vehicle control architectures include decentralized control, hierarchical control, and centralized control. Among them, the decentralized control architecture is the most developed and widely applied. Its control principle involves collecting vehicle state data through independent sensors of each sub-control system, with subsystem controllers outputting control commands to the actuators based on their respective control goals. The advantage of decentralized control architecture lies in its low system complexity, but its disadvantages are non-unified software development platforms, serious difficulty in function integration, deeply bound control strategies to hardware causing new features to conflict, and sensor information not being reusable, leading to waste. The decentralized control architecture can only achieve simple integrated control. Under extreme conditions, the high coupling degree of steering and braking systems means that the decentralized architecture does not achieve true longitudinal and lateral dynamics decoupling and cannot reduce conflicts between system control functions [7,8,9]. In a centralized control system, various sensors and observers are utilized as required to gather vehicle data for a central controller, facilitating information sharing. The controller devises globally coordinated control strategies or optimization algorithms, directly transmitting execution signals to all subsystem actuators. Its advantages lie in eliminating redundant hardware installations and achieving globally optimal control performance; however, it requires fault-tolerant design for the central controller, with reliability still needing improvement [10,11]. The hierarchical centralized control architecture comprises the state input, coordination decision, and control execution layers. The state input layer receives driving commands, defines driving situations, and categorizes operational intents. The coordination decision layer selects coordination strategies based on the operational mode and allocates control inputs to chassis subsystems. The control execution layer includes controllers for each subsystem, which are responsible for implementing control commands and transmitting signals to actuators. Its advantages include functional decoupling, ease of expansion, and robustness to local faults, but it cannot achieve globally optimal control [12,13,14]. In summary, compared with centralized architecture, hierarchical control architecture has the advantages of high fault tolerance and low complexity. Compared with decentralized architecture, it benefits from independent decoupling between subsystems, facilitating system development and function expansion. Therefore, it is the ideal architecture for current vehicle motion control strategies.

The core objective of vehicle motion control strategies is to ensure the safety of vehicle operation, especially stability in complex scenarios. Extensive research has been conducted on vehicle control strategies regarding both longitudinal and lateral stability. Longitudinal stability can be divided into two dimensions: driving and braking. In the braking scenario, the objective of vehicle motion control is to enhance braking efficiency and directional stability during braking, with key factors affecting both the distribution of braking force and control of wheel slip ratio. Traditional braking force distribution relies on fixed proportional coefficients between the front and rear axles. While this method is logically simple and does not require complex distribution algorithms, its drawback lies in its inability to adapt to the varying distribution needs of vehicles across different scenarios. A typical function for slip ratio control is an anti-lock braking system (ABS) [15,16,17,18]. ABS adjusts brake torque by setting slip ratio and angular acceleration thresholds and achieves steering compensation and brake coordination through yaw rate thresholds. Its advantages lie in simplicity and independence from state observation, but it lacks robustness and overly relies on calibration, affecting driving comfort [19,20]. Some scholars have implemented ABS using slip mode control, taking actual and optimal slip ratios as inputs, designing a slip surface to output braking torque, and adjusting the actual slip ratio to the target. The advantages include adjustable slip mode, minimal parameter adjustment, and fast response. However, improper parameter selection in nonlinear time-varying systems can lead to oscillations, affecting stability [21,22,23,24]. In a driving scenario, the main factor affecting driving safety is the acceleration slip regulation (ASR). Some researchers use PID control to achieve anti-slip by tracking the optimal slip ratio of the wheels. This method adjusts motor or braking torque under different road conditions. Its advantages are simplicity and wide engineering application, but it has low control precision and is prone to steady-state errors [25,26]. Some researchers use fuzzy control logic, taking the accelerator pedal position and the slope of the tire/road adhesion coefficient as inputs to output the required motor torque to achieve ASR. This method is simple in principle and does not require an accurate model, but it has extensive theoretical research and is difficult to implement in actual controllers [27,28,29,30]. In the lateral stability of vehicles, two key aspects need to be considered. First, the identification of lateral instability. Some scholars use phase plane diagrams to categorize vehicle states and apply various DYC control logic to enhance lateral stability during steering. However, the variability in the vehicle environment and driver style makes it challenging for the phase plane to determine stable regions accurately and swiftly [31]. The next aspect is the control of lateral stability, which includes active front steering (AFS) and direct yaw moment control systems (DYCs). The objectives of these control strategies are to ensure vehicle stability during lateral motion, thereby enhancing driving safety and comfort [32,33,34]. Some scholars have designed AFS and DYC systems based on a Multi-Agent Systems (MAS) distributed integrated control architecture, ensuring efficient communication and coordination among components of electric vehicles, enabling real-time adjustments, and enhancing overall performance and response speed. AFS and DYC optimize vehicle dynamic performance and safety [35,36]. Some scholars have utilized Fuzzy Time Series Modeling (FTSM) and Second Order Sliding Mode Control (SOSM) techniques to implement AFS and DYC for vehicles. FTSM is used for fuzzy modeling, and SOSM is used for real-time control. The advantage lies in the system’s adaptability to various environments and road conditions, yet it depends on sensor accuracy and computational complexity, facing challenges in real-time responsiveness [37,38].

Existing chassis-integrated control methods have conducted extensive research on lateral and longitudinal stability control, effectively improving control performance, but multidimensional stability interference issues still exist [39,40]. Based on this, this paper proposes a lateral and longitudinal stability control system with double-layer torque distribution based on nonlinear model predictive control (NMPC). This strategy reintegrates the control structure of yaw stability into the control objectives of longitudinal stability, achieving effective lateral and longitudinal stability control through the combined action of multiple controllers. First, based on the state observation of sensor signals, the ideal motion state of the vehicle is obtained. Second, based on NMPC, lateral and longitudinal controllers are developed to obtain the vehicle control quantities for tracking the ideal motion state. Finally, the control instructions issued by the actual controllers are sent to the actuators to optimize the vehicle’s stability performance. The advantages of this work include the following points:

1. The integrated control strategy of dual torque distribution consists of a lateral stability controller and a longitudinal stability controller. The dual-layer design can avoid the mutual effect of the lateral controller and the longitudinal controller. By reallocating the control results of the lateral controller within the longitudinal stability controller, the vehicle’s longitudinal and lateral stability is enhanced.
2. The proposed strategy achieves an integrated control against both lateral deviation and longitudinal slip, showing a positive impact on the coordinated control of lateral and longitudinal stability.
3. Based on the distributed control architecture of EMB, independent torque control is performed according to the current state of each wheel, achieving better results compared with the previous centralized control architecture. Dynamic control of driving and braking torque for each wheel is developed based on MPC, ensuring that the slip rates of each wheel approach the target slip rate and that the vehicle stability parameters remain within a reasonable range.

The remainder of this paper is structured as follows: Section 2 illustrates the vehicle model, and Section 3 discusses the integrated control strategy design. Simulations are provided in Section 4. The conclusions are presented in Section 5.

## 2. Vehicle and Component Model

**Figure 1:**
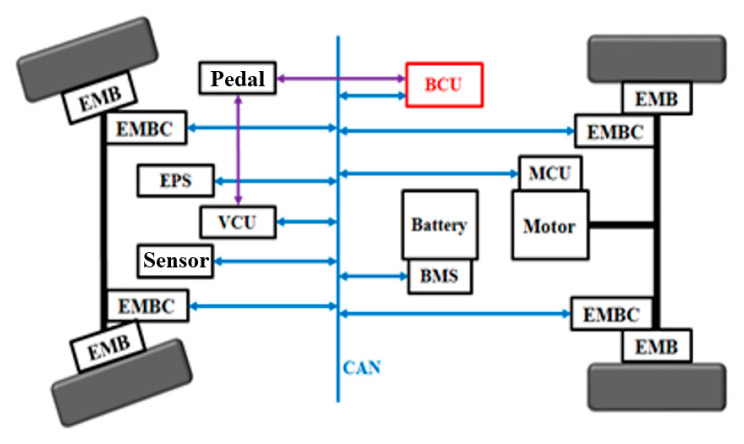
Automotive electronic control system architecture.

This paper focuses on an electric commercial vehicle as the research subject, with its structure shown in Figure 1. The studied vehicle includes a single-drive motor for the rear axle, a power battery, a gearbox, and a main reducer. The EMB acts as the brake system actuator, controlled by the Brake Control Unit. The Brake Control Unit communicates via the Controller Area Network (CAN) bus, connecting to the EMB motor control unit and the Vehicle Control Unit. The vehicle parameters are shown in Table 1.

### 2.1. Vehicle Model

During the vehicle operation, the power source should provide the tractive force that overcomes the resistance of the vehicle in order to drive the studied vehicle. The various resistances include air resistance and acceleration resistance, and the corresponding relationship between the tractive force and the resistance is formulated as:

(1) $$\frac{T_{tq}i_{0}i_{g}\eta_{t}}{r}=\frac{1}{2}C_{D}A\rho v^{2}+Mgf+\delta M{\dot{v}}_{x}$$

where M denotes vehicle mass, $v_{x}$ denotes vehicle longitudinal velocity, $T_{tq}$ denotes motor torque, $\eta_{t}$ denotes transmission efficiency, r denotes tire rolling radius, $C_{d}$ denotes air resistance coefficient, A denotes the windward area, ρ denotes air density, f denotes road rolling resistance coefficient, $\delta =1+\frac{1}{M}\frac{\sum I_{w}}{r^{2}}+\frac{1}{M}\frac{I_{f}i_{g}^{2}i_{0}^{2}\eta_{t}}{r^{2}}$ denotes the rotational mass coefficient, $i_{0}$ denotes the main reduction gear ratio, $i_{g}$ denotes the vehicle’s transmission ratio. $I_{w}$ denotes the rotating inertia of the wheels, $I_{f}$ denotes the rotating inertia of the motor.

### 2.2. Response Characteristics of the EMB Brake

**Figure 2:**
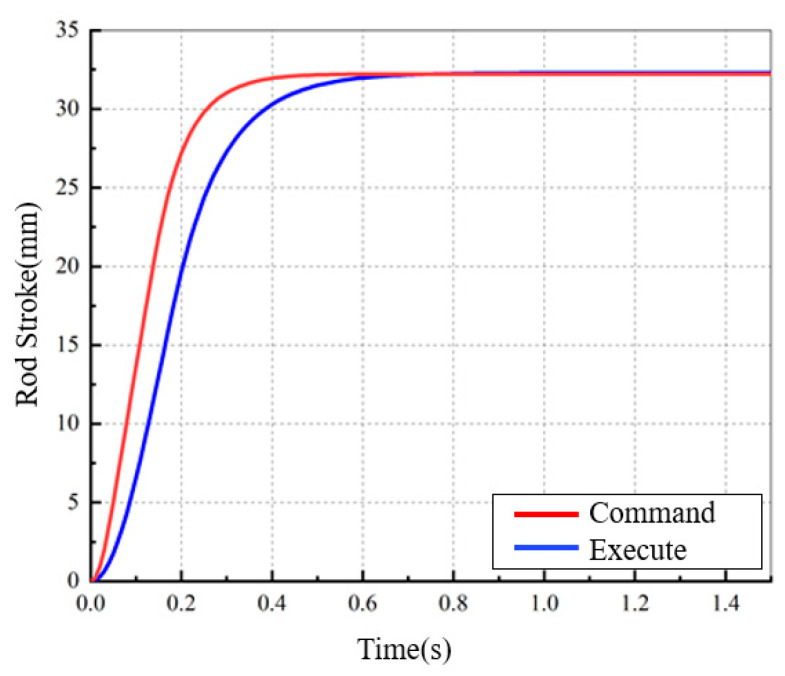
EMB Response Characteristics.

EMB achieves brake control through a motor-driven transmission mechanism and a clamping brake disc, thereby decoupling the brake pedal from the braking torque at the mechanical level. Additionally, compared with pneumatic brake systems, EMB offers advantages such as fast response, high control accuracy, and low maintenance costs. The response characteristics of EMB are shown in Figure 2.

### 2.3. Tire Model

The Magic Formula tire model is an ideal tire model based on experimental data characterized by high fitting accuracy. Therefore, it is suitable for fields requiring precise descriptions of tire dynamics, such as product design, vehicle dynamic simulation, and experimental comparison. In this paper, the Magic Formula is used to calculate tire forces, which can be expressed as:

(2) $$\left\{\begin{array}{l} Y\left(x\right)=y\left(x\right)+S_{v}\\ y=D\cdot \sin\left\{C\cdot \arctan\left[B\cdot x-E\cdot \left(B\cdot x-\arctan B\cdot x\right)\right]\right\}\\ x=X+S_{h} \end{array}\right.$$

In this formula, Y represents lateral force, longitudinal force, or aligning torque, X represents the slip angle or longitudinal slip ratio, D denotes the peak factor, B denotes the stiffness factor, E denotes the curvature factor, C denotes the shape factor, $S_{h}$ denotes the horizontal shift, and $S_{v}$ denotes the vertical curve shift.

### 2.4. Drive Motor

The motor serves as the power source for vehicles, driving the vehicle and regulating wheel slip. The output power of the motor $P_{em}$ in this paper can be represented as:

(3) $$P_{em}=\frac{\pi }{30}\cdot n_{em}\cdot T_{em}$$

where $T_{em}$ represents the output torque of the motor and $n_{em}$ represents the speed of the motor. According to the test parameters, the motor efficiency $\eta_{M}$ can be determined based on motor speed $n_{M}$ and motor torque $T_{M}$.

(4) $$\eta_{M}=\eta_{M}\left(n_{M},T_{M}\right)$$

Regarding the impact on motor torque response characteristics, the motor torque is modeled as a first-order response, according to the simulation step size $\tau_{s}$, which can be expressed as:

(5) $$T_{em\_cmd}=T_{em}+\tau_{s}\cdot {\dot{T}}_{em}$$

In this formula, $T_{em\_cmd}$ is the required motor torque. During vehicle driving, the motor torque is transmitted to the two driving wheels through the transmission, driveshaft, final reducer, and half-shaft. The motor torque $T_{ele}$ acting on the drive wheels can be calculated as:

(6) $$T_{ele}=\frac{i_{g}\cdot i_{0}}{2}\cdot T_{em}$$

In the formula, $i_{g}$ represents the transmission ratio and $i_{0}$ represents the final drive ratio.

## 3. Control Strategy

**Figure 3:**
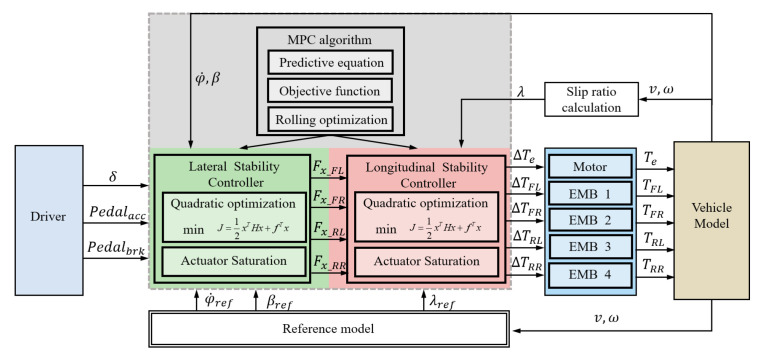
Integrated control system architecture.

In this section, a chassis dynamics integrated control system utilizing dual torque distribution is proposed. This control system integrates the functions of the ASR, ABS, and DYC. The control strategy is illustrated in Figure 3.

For a vehicle being controlled, the actual driving and braking demands can be identified from the driver’s operation intentions. The obtained driving and braking demands are the overall torque requirements of the vehicle. This section uses model predictive control to design a comprehensive stability controller, which is divided into two stages: lateral stability control and longitudinal stability control. Lateral stability control intervenes in the stability of lateral conditions such as turning. The primary torque output from this control serves as the control input for the subsequent longitudinal stability control, achieving comprehensive control of both lateral and longitudinal stability.

### 3.1. Basic Derivation of Model Predictive Control

Model predictive control has outstanding advantages in solving multivariable constrained optimization control problems and is widely used in the industry. To effectively track the reference values of actual system quantities, the control strategy proposed in this paper uses model predictive control to design a comprehensive stability controller. The solution to the model predictive control problem first depends on the state-space equations describing the linear or nonlinear system functions:

(7) x(k+1)=f(x(k),u(k))

where x(k) represents the state of the system at time t=k; u(k) represents the control input at time t=k; d(k) is the measurable external disturbance variable. For the state and control variables at time t, $x_{0}(k+1)=f(x_{0}(k),u_{0}(k))$. A general system state-space function is usually written in the form of Equation (8).

(8) x(k+1)=Ax(k)+Bu(k)+d(k)

where:

$$A=\frac{\partial f}{\partial x}{|}_{\left(x_{0}(k),u_{0}(k)\right)},B=\frac{\partial f}{\partial u}{|}_{\left(x_{0}(k),u_{0}(k)\right)},d(k)=f(x_{0}(k),u_{0}(k))-Ax_{0}(k)-Bu_{0}(k)$$

Set the prediction horizon of the model predictive control to be *N*, and the control horizon to be m, with m≤N. To derive the system predictive equation, the following assumption is made: the measurable disturbance remains constant within the prediction horizon *N*, d(k)=d(k+i),i=1,2,…,N−1. Then we have:

(9) $$\begin{array}{l} x(k|k)=x(k)\\ x(k+1|k)=Ax(k|k)+Bu(k|k)+d(k)\\ \;\;\;\;\;\;=Ax(k)+Bu(k|k)+d(k)\\ x(k+2|k)=Ax(k+1|k)+Bu(k+1|k)+d(k)\\ \;\;\;\;\;\;=A^{2}x(k)+ABu(k|k)+Bu(k+1|k)+Ad(k)+d(k)\\ x(k+N|k)=A^{N}x(k)+A^{\left(N-1\right)}Bu(k|k)+\ldots +Bu(k+N-1|k)+A^{\left(N-1\right)}d(k)+Ad(k)+d(k) \end{array}$$

In the above equation, $x\left(k+N\left|k\right.\right)$ represents the prediction at time t=k for time t=k+N. The *N*-step output vector and the corresponding control vector can be defined as follows:

(10) $$X_{k}=\left[\begin{array}{c} x\left(k\left|k\right.\right)\\ x\left(k+1\left|k\right.\right)\\ x\left(k+2\left|k\right.\right)\\ \vdots \\ x\left(k+N\left|k\right.\right) \end{array}\right]$$

(11) $$U_{k}=\left[\begin{array}{c} u\left(k\left|k\right.\right)\\ u\left(k+1\left|k\right.\right)\\ u\left(k+2\left|k\right.\right)\\ \vdots \\ u\left(k+N-1\left|k\right.\right) \end{array}\right]$$

The output prediction for the next N steps of the system can be calculated using the predictive equation from Equation (12):

(12) $$X_{k}=Mx_{k}+CU_{k}+D$$

where:

(13) $$M=\left[\begin{array}{c} I\\ A\\ A^{2}\\ \vdots \\ A^{N} \end{array}\right]$$

(14) $$C=\left[\begin{array}{cccc} 0 & 0 & \cdots & 0\\ B & 0 & \cdots & 0\\ AB & B & \cdots & 0\\ \cdots & \cdots & \cdots & 0\\ A^{N-1}B & A^{N-2}B & \cdots & B \end{array}\right]$$

(15) $$D=\left[\begin{array}{c} 0\\ d\left(k\right)\\ Ad\left(k\right)+d\left(k\right)\\ \vdots \\ A^{N-1}d\left(k\right)+\cdots +Ad\left(k\right)+d\left(k\right) \end{array}\right]$$

For a general system, the cost function usually includes the difference between the state variable and the reference value, as well as minimizing the control input. The difference between the state variable and the reference value is written as $E\left(\left.k+i\right|k\right)=x\left(\left.k+i\right|k\right)-r\left(\left.k+i\right|k\right)$, therefore, the cost function can be written as:

(16) $$J\left(x\left(k\right),U\left(k\right)\right)=\sum_{i=0}^{N}\left({\left(E\left(\left.k+i\right|k\right)\right)}^{T}QE\left(\left.k+i\right|k\right)+{\left(u\left(\left.k+i\right|k\right)\right)}^{T}Ru\left(\left.k+i\right|k\right)\right)$$

where Q is the state tracking matrix coefficient, and R is the control matrix coefficient. To obtain the control input that effectively tracks the reference state, the cost function needs to be minimized, that is:

(17) $$\min J\left(x\left(k\right),U\left(k\right)\right)$$

Quadratic programming can be used to minimize the cost function and obtain the optimal solution for model predictive control. The optimal control sequence of the control problem obtained at time t is u*, and the first element of the optimal control sequence is applied to the system,

(18) $$u*\left(k\right)=\left[1,0,\cdots ,0\right]U_{k}\left(k\right)$$

### 3.2. Integrated Control Strategy Design

#### 3.2.1. Lateral Stability Control Strategy

To complete the design of the integrated control strategy, it is first necessary to provide system parameters describing lateral and longitudinal dynamics. This paper selects the slip angle and yaw rate as the evaluation indicators for lateral stability and the slip ratio as the evaluation indicator for longitudinal stability. During vehicle steering, the lateral force may cause the vehicle to have a tendency to understeer or oversteer, which can lead to severe vehicle instability. The lateral stability of the vehicle is measured by the yaw rate $\dot{\varphi }$ and the slip angle β. To effectively reduce the yaw amplitude of the vehicle, a common approach is to apply braking to the wheels, causing the vehicle to generate a yaw moment in the opposite direction. To include the coordination control variable $F_{x}$, the equation is written as Equation (19):

(19) $$\left\{\begin{array}{l} I_{z}\ddot{\varphi }=ak_{1}\left(\beta +\frac{a\dot{\varphi }}{v_{x}}-\delta \right)-bk_{2}\left(\beta -\frac{b\varphi }{v_{x}}\right)+\left(F_{x,FR,cmd}-F_{x,FL,cmd}\right)\frac{B_{1}}{2}+\left(F_{x,RR,cmd}-F_{x,RL,cmd}\right)\frac{B_{2}}{2}\\ M\left({\dot{v}}_{y}+v_{x}\dot{\varphi }\right)=k_{1}\left(\beta +\frac{a\dot{\varphi }}{v_{x}}-\delta \right)+k_{2}\left(\beta -\frac{b\varphi }{v_{x}}\right) \end{array}\right.$$

where $I_{z}$ represents the moment of inertia of the car around the *z* axis; φ represents the yaw angle; β represents the slip angle; δ represents the front wheel steering angle; $k_{1}$ and $k_{2}$ represent the cornering stiffness of the front and rear axle tires, a represents the distance from the vehicle center of mass to the front axle, and b represents the distance from the vehicle center of mass to the rear axle.

The vehicle slip angle can be calculated by Equation (20):

(20) $$\beta =\frac{v_{y}}{v_{x}}$$

The derivative of the slip angle is:

(21) $$\dot{\beta }=\frac{{\dot{v}}_{y}}{v_{x}}-\frac{{\dot{v}}_{x}\beta }{v_{x}}$$

In general, the slip angle of the vehicle is very small during steering, and there are no significant speed fluctuations during steering. ${\dot{v}}_{x}\beta \ll v_{x}$. Therefore, it can be approximated as $\dot{\beta }=\frac{{\dot{v}}_{y}}{v_{x}}$.

Then, Equation (19) can be expressed as:

(22) $$M\dot{\beta }v_{x}=-Mv_{x}\dot{\varphi }+k_{1}\left(\beta +\frac{a\dot{\varphi }}{v_{x}}-\delta \right)+k_{2}\left(\beta -\frac{b\dot{\varphi }}{v_{x}}\right)$$

Rewrite the above equation into a continuous state-space expression:

(23) $$\left[\begin{array}{c} \ddot{\varphi }\\ \dot{\beta } \end{array}\right]=\left[\begin{array}{cc} \frac{a^{2}k_{1}+b^{2}k_{2}}{I_{z}v_{x}} & \frac{ak_{1}-bk_{2}}{I_{z}}\\ \frac{ak_{1}-bk_{2}}{Mv_{x}^{2}}-1 & \frac{k_{1}+k_{2}}{Mv_{x}} \end{array}\right]\left[\begin{array}{c} \dot{\varphi }\\ \beta \end{array}\right]+\left[\begin{array}{cccc} -\frac{B_{1}}{2I_{z}} & \frac{B_{1}}{2I_{z}} & -\frac{B_{2}}{2I_{z}} & \frac{B_{2}}{2I_{z}}\\ 0 & 0 & 0 & 0 \end{array}\right]\left[\begin{array}{c} F_{x,FL,cmd}\\ F_{x,FR,cmd}\\ F_{x,RL,cmd}\\ F_{x,RR,cmd} \end{array}\right]+\left[\begin{array}{c} -\frac{ak_{1}}{I_{z}}\\ -\frac{k_{1}}{Mv_{x}} \end{array}\right]\delta$$

Equation (23) is linear, referring to the state Equation (8), we can obtain:

(24) $$A=\left[\begin{array}{cc} \frac{a^{2}k_{1}+b^{2}k_{2}}{I_{z}v_{x}} & \frac{ak_{1}-bk_{2}}{I_{z}}\\ \frac{ak_{1}-bk_{2}}{Mv_{x}^{2}}-1 & \frac{k_{1}+k_{2}}{Mv_{x}} \end{array}\right]$$

(25) $$B=\left[\begin{array}{cccc} -\frac{B_{1}}{2I_{z}} & \frac{B_{1}}{2I_{z}} & -\frac{B_{2}}{2I_{z}} & \frac{B_{2}}{2I_{z}}\\ 0 & 0 & 0 & 0 \end{array}\right]$$

(26) $$d\left(t\right)=\left[\begin{array}{c} -\frac{ak_{1}}{I_{z}}\\ -\frac{k_{1}}{Mv_{x}} \end{array}\right]\delta$$

Among them $x\left(t\right)={\left[\begin{array}{cc} \dot{\varphi } & \beta \end{array}\right]}^{T}$, $u\left(t\right)={\left[\begin{array}{cccc} F_{x,FL,cmd} & F_{x,FR,cmd} & F_{x,RL,cmd} & F_{x,RR,cmd} \end{array}\right]}^{T}$; A, B are the coefficient matrices of state variables and control inputs, and d is the error between nonlinear systems and linear time-varying systems. The reference values of state parameters describe the ideal state of vehicle motion, and the main goal of the control strategy is to ensure that key parameters of vehicle stability always efficiently track the ideal state. To ensure that the vehicle has sufficient steering capability and good lateral stability, the lateral stability parameters adopt a dynamic calculation formula. The lateral stability parameters, referring to the yaw rate and the center of gravity lateral deviation angle, can be calculated as follows:

(27) $${\left[\begin{array}{c} \ddot{\varphi }\\ \dot{\beta } \end{array}\right]}_{ref}=A_{ref}{\left[\begin{array}{c} \dot{\varphi }\\ \beta \end{array}\right]}_{ref}+B_{ref}\delta$$

where:

(28) $$A_{ref}=\left[\begin{array}{cc} \frac{bk_{2}-ak_{1}}{I_{z}} & -\frac{a^{2}k_{1}+b^{2}k_{2}}{I_{z}v_{x}}\\ -\frac{k_{1}+k_{2}}{Mv_{x}} & \frac{bk_{2}-ak_{1}}{Mv_{x}^{2}}-1 \end{array}\right]$$

(29) $$B_{ref}=\left[\begin{array}{c} \frac{ak_{1}}{I_{z}}\\ \frac{k_{1}}{Mv_{x}} \end{array}\right]$$

Considering the tracking control effect of lateral stability and the amplitude of control input variables, we designed an optimization solver for Equation (30):

(30) $$\begin{array}{l} J_{stability\_lateral}=\sum_{i=0}^{N}({\left\|\varphi (k+i\left|k\right.)-\varphi_{ref}(k+i\left|k\right.)\right\|}_{Q_{\varphi }}+{\left\|\beta (k+i\left|k\right.)-\beta_{ref}(k+i\left|k\right.)\right\|}_{Q_{\beta }})\\ \;\;\;\;\;\;+\sum_{i=0}^{N}{\left\|u(k+i\left|k\right.)\right\|}_{R_{\varphi ,\beta }} \end{array}$$

The minimization of Equation (30) is subject to the following state and input constraints:

(31) $$s.t.\left\{\begin{array}{c} \varphi_{\min}<\varphi <\varphi_{\max}\\ \beta_{\min}<\beta <\beta_{\max}\\ F_{x,i,\min}<F_{x,i,cmd}<F_{x,i,\max} \end{array}\right.$$

When the vehicle is turning and is already unstable or trending towards instability, effective control of vehicle stability can be achieved by applying braking forces to generate yaw moments based on the difference between the actual and reference values of the target parameters. There are basically two methods: single-wheel braking and one-sided wheel braking. Through analysis, it was found that single-wheel braking control can generate larger yaw moments with the same braking torque, thus achieving more effective control of the vehicle stability. This paper achieves control of cornering stability through single-wheel braking. In order to determine the specific braking wheels under different cornering conditions, target braking wheel decision rules, as shown in Table 2, are established.

It is stipulated that when the steering wheel turns left δ>0, it turns right δ<0. When the yaw rate is clockwise $\dot{\varphi }>0$, it is counterclockwise $\dot{\varphi }<0$.

#### 3.2.2. Longitudinal Stability Control Strategy

The longitudinal stability of the vehicle mainly considers the vehicle slip. When not turning, the stability of the vehicle mainly depends on the vehicle slip state. When the vehicle needs to turn, the stability of the vehicle should be comprehensively considered between yaw and slip. Therefore, reintegrating the control input results of lateral stability into the longitudinal stability controller helps to solve the coordination problem between lateral and longitudinal directions.

This paper mainly considers the influence of the longitudinal slip ratio on tire forces. The longitudinal slip ratio refers to the proportion of sliding components in vehicle starting and braking conditions. When the vehicle travels at idle speed on a road with good adhesion, the interaction between the wheel and the ground should be approximately pure rolling friction, and the slip ratio approaches 0. During starting and braking, sliding friction may occur, resulting in a larger slip ratio. When the wheels are completely locked, the slip ratio should be 1. The longitudinal stability controller uses Equation (32) to calculate the slip ratio of vehicle driving and braking:

(32) $$\lambda_{i}=\left\{\begin{array}{r} \begin{array}{cc} \frac{\omega_{i}r_{i}-v_{x}}{\omega_{i}r_{i}} & ,v_{x}<\omega_{i}R_{i} \end{array}\\ \begin{array}{cc} \frac{v_{x}-\omega_{i}r_{i}}{v_{x}} & ,v_{x}>\omega_{i}R_{i} \end{array} \end{array}\right.$$

Among them, $v_{x}$ is the vehicle’s longitudinal speed, $\omega_{i}$ is the wheel angular velocity, and i can be FL, FR, RL, or RR. During driving, the wheel speed $\omega_{i}R_{i}$ is greater than the vehicle longitudinal speed $v_{x}$; during braking, the vehicle longitudinal speed $v_{x}$ is greater than the wheel speed $\omega_{i}$. The time derivative of $\lambda_{i}$ is:

(33) $${\dot{\lambda }}_{i}=\left\{\begin{array}{l} \begin{array}{cc} \frac{{\dot{\omega }}_{i}v_{x}R_{i}-{\dot{v}}_{x}\omega_{i}R_{i}}{{\left(\omega_{i}R_{i}\right)}^{2}} & ,v_{x}<\omega_{i}R_{i} \end{array}\\ \begin{array}{cc} \frac{{\dot{v}}_{x}\omega_{i}R-{\dot{\omega }}_{i}v_{x}R_{i}}{{v_{x}}^{2}} & ,v_{x}>\omega_{i}R_{i} \end{array} \end{array}\right.$$

The derivative of the vehicle’s longitudinal speed can be calculated by the following equation:

(34) $${\dot{v}}_{x}={\left(-1\right)}^{k}\frac{F_{x,i}}{M_{x,i}}$$

Among them, $F_{x,i}$ is the longitudinal force at each wheel, $M_{x,i}$ is the vertical load at each wheel, and k is the parameter representing driving and braking, with k=0 when driving and k=1 when braking. The rotational dynamics of the wheels should include the actual control variables of the controller, which can be expressed as:

(35) $$\dot{\omega }=\frac{{\left(-1\right)}^{k}\left[\left(T_{x,i}-\Delta T_{x,i}\right)-F_{x,i}R_{x,i}\right]}{I}$$

Among them, I represents the wheel moment of inertia; $T_{x,i}$ represents the braking torque at each wheel; $\Delta T_{x,i}$ represents the compensating braking torque applied by the MPC controller. The longitudinal force at each wheel is calculated using the Magic Formula. Substituting the above equations into Equation (33), we obtain the following state-space equation for wheel slip ratio:

(36) $${\dot{\lambda }}_{i}=\left\{\begin{array}{l} \frac{R_{i}\left(T_{x,i}-\Delta T_{x,i}\right){\left(1-\lambda_{i}\right)}^{2}}{Iv_{x}}-\frac{{R_{i}}^{2}F_{x}\left[{\left(1-\lambda_{i}\right)}^{2}+I\left(1-\lambda_{i}\right)/\left(M_{x,i}{R_{i}}^{2}\right)\right]}{Iv_{x}}\;v_{x}<\omega_{i}R_{i}\\ \frac{R_{i}\left(T_{x,i}-\Delta T_{x,i}\right)}{Iv_{x}}-\frac{{R_{i}}^{2}F_{x}\left[1+I\left(1-\lambda_{i}\right)/\left(M_{x,i}{R_{i}}^{2}\right)\right]}{Iv_{x}}\;v_{x}\geq \omega_{i}R_{i} \end{array}\right.$$

The rate of change in slip ratio ${\dot{\lambda }}_{i}$ is a nonlinear equation of $\lambda_{i}$, $\Delta T_{x,i}$ which can be written as:

(37) $${\dot{\lambda }}_{i}=f_{v}\left(\lambda_{i},\Delta T_{x,i}\right)$$

This equation is a nonlinear equation, and discretizing it yields:

(38) $$\lambda_{i}\left(k+1\right)=f\left(\lambda_{i}\left(k\right),\Delta T_{x,i}\left(k\right)\right)$$

where $\lambda_{i}(k)$ is the slip ratio at the current time step, and $\lambda_{i}\left(k+1\right)$ is the slip ratio at the next time step. For the longitudinal stability controller, in the state equation of the reference model predictive control Equation (8), x(t) is the controller state variable, u(t) is the controller control input, $x={\left[\lambda_{FL},\lambda_{FR},\lambda_{RL},\lambda_{RR}\right]}^{T}$, $u={\left[\Delta T_{FL},\Delta T_{FR},\Delta T_{RL},\Delta T_{RR}\right]}^{T}$. *A* and *B* are coefficient matrices of the state and control variables, with specific values given in Equations (39) and (40).

(39) $$A=\left\{\begin{array}{c} \left(\begin{array}{l} 1-\frac{2\Delta t\cdot R_{i}(T_{x,i}-\Delta T_{x,i})(1-\lambda_{i})}{Iv_{x}}-\frac{\Delta t\cdot {R_{i}}^{2}}{Iv_{x}}\left[{F_{x}}^{\prime }{\left(1-\lambda_{i}\right)}^{2}-2F_{x}(1-\lambda_{i})\right]\\ -\frac{\Delta t}{M_{x,i}v_{x}}\left[{F_{x}}^{\prime }(1-\lambda_{i})-F_{x}\right] \end{array}\right)\;v_{x}<\omega_{i}R_{i}\\ 1+\frac{\Delta t\cdot {F_{x}}^{\prime }}{M_{x,i}v_{x}}\lambda_{i}+\frac{\Delta t\cdot F_{x}}{M_{x,i}v_{x}}+\left(\frac{\Delta t\cdot {R_{i}}^{2}}{Iv_{x}}+\frac{\Delta t}{M_{x,i}v_{x}}\right){F_{x}}^{\prime }\;v_{x}>\omega_{i}R_{i} \end{array}\right.$$

(40) $$B=\left\{\begin{array}{c} -\frac{\Delta t\cdot R_{i}{\left(1-\lambda_{i}\right)}^{2}}{Iv_{x}}\begin{array}{cc} & v_{x}<\omega_{i}R_{i} \end{array}\\ -\frac{\Delta t\cdot R_{i}}{Iv_{x}}\begin{array}{cc} & v_{x}>\omega_{i}R_{i} \end{array} \end{array}\right.$$

(41) $$F_{x}=D\cdot \sin\left\{C\cdot \arctan\left[B\cdot x-E\cdot \left(B\cdot x-\arctan B\cdot x\right)\right]\right\}$$

(42) $$\begin{array}{c} {F_{x}}^{\prime }=D\cdot \cos\left\{C\arctan\left[B\lambda_{i}-E(B\lambda_{i}-\arctan B\lambda_{i})\right]\right\}\\ \cdot \frac{C}{1+{\left[B\lambda_{i}-E(B\lambda_{i}-\arctan B\lambda_{i})\right]}^{2}}(B-EB+\frac{EB}{1+{\left(B\lambda_{i}\right)}^{2}}) \end{array}$$

$F_{x}$ is the longitudinal force of the tire and ${F^{\prime }}_{x}$ is the derivative of the longitudinal force of the tire; d is the error between the nonlinear system and the linear time-varying system, $d\left(k\right)=f\left(x_{0}\left(k\right),u_{0}\left(k\right)\right)-Ax_{0}\left(k\right)-Bu_{0}\left(k\right)$.

The control input is the change in the output torque of the wheel-side actuator. Since the vehicle used in this paper is driven by a single motor on the rear axle, when the vehicle is in the driving state, according to Equation (6), we have:

(43) $$T_{ele}=T-\Delta T_{i}$$

After obtaining the change in the wheel-side drive torque, the change in the actual motor drive torque can also be obtained by combining it with Equation (6).

The tracking objective of the longitudinal stability state parameters of the vehicle is still to make them track the reference values $\lambda_{ref}$ as closely as possible. The reference slip ratio can be obtained through the relationship with the road adhesion, and the slip ratio under different road adhesions can be described by Equation (44).

(44) $$\mu (\lambda )=c_{1}(1-e^{-c_{2}\lambda })-c_{3}\lambda$$

**Figure 4:**
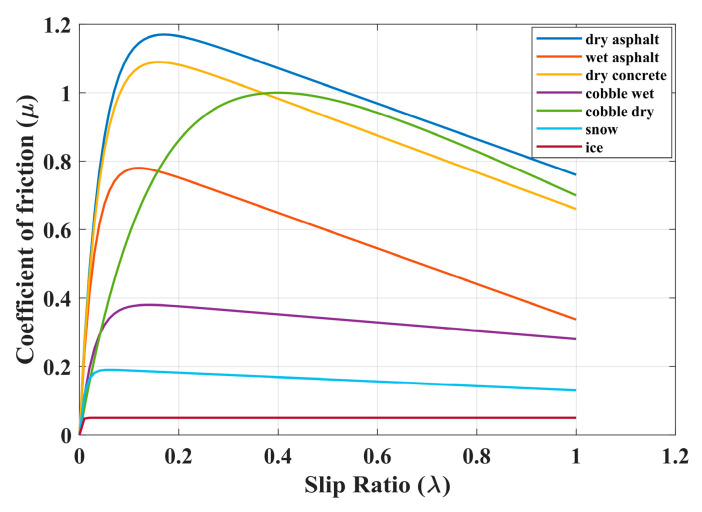
Friction coefficient as a function of slip ratio for different surfaces.

According to the parameters provided in Table 3, eight curves in Figure 4 can be obtained [41].

The cost function for longitudinal stability can be written as:

(45) $$\begin{array}{l} J_{stability\_longitudinal}=\sum_{i=0}^{N-1}({\left\|\lambda (k+i\left|k\right.)-\lambda_{ref}(k+i\left|k\right.)\right\|}_{Q_{\lambda }})+\sum_{i=0}^{N}{\left\|u(k+i\left|k\right.)\right\|}_{R_{\lambda }}\\ \;\;\;\;\;\;\;+{\left\|\lambda (k+N\left|k\right.)-\lambda_{ref}(k+N\left|k\right.)\right\|}_{F} \end{array}$$

The minimization of Equation (45) is subject to the following state constraints:

(46) $$s.t.\left\{\begin{array}{c} \lambda_{\min}<\lambda <\lambda_{\max}\\ T_{x,i,\min}<T<T_{x,i,\max}\\ \Delta T_{x,i,\min}<\Delta T_{x,i,cmd}<\Delta T_{x,i,\max} \end{array}\right.$$

## 4. Model-in-the-Loop Test Results and Analysis

In this section, the stability of the NMPC-based integrated control strategy proposed in this paper has been verified in terms of both lateral and longitudinal stability. The proposed strategy integrates the functionalities of ABS, ASR, and DYC. To thoroughly test the advantages of the proposed strategy, it is necessary to validate each of their functionalities separately. In particular, the performance of ABS and ASR is tested under longitudinal driving and braking conditions. For the coordinated control in both lateral and longitudinal stability, the performance is verified effectively for double-lane changes.

To assess the longitudinal stability of the vehicle, braking and driving conditions are designed to observe the vehicle’s performance on different roads. In the braking condition, a comparison is made with the rule-based ABS control strategy. In driving conditions, the vehicle equipped with the proposed integrated control strategy is compared with a vehicle without traction control to verify the effectiveness of this strategy. To assess the coordinated lateral and longitudinal stability of the vehicle, a double-lane change test group is designed. The test group compares vehicles under the control strategy proposed in this paper with vehicles without control to verify the effectiveness of the integrated control strategy in terms of lateral stability in terms of coordinated lateral and longitudinal stability.

The above-mentioned rule-based ABS control strategy relies on wheel angular acceleration and slip ratio thresholds to increase and decrease braking force. The rule-based ABS control strategy achieves anti-lock braking by controlling the pressure increase and decrease in each wheel brake.

For these validations, Matlab/Simulink is employed to verify the effectiveness of the control strategy. The vehicle parameters are listed in Table 1. The simulation was performed using Matlab 2021b, and the model ran on a personal computer equipped with an Intel i7-13700H processor and 16 GB of memory. All simulations are run with a fixed step size of 0.01 s.

### 4.1. Simulation of Braking Conditions for the Integrated Control Strategy

**Figure 5:**
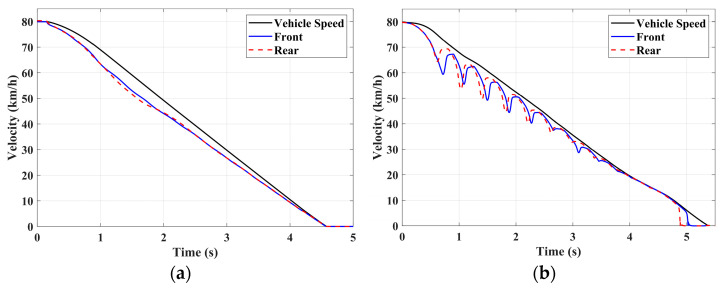
High-adhesion braking test: vehicle speed and wheel speed. (**a**) Integrated control (**b**) Rule-based ABS strategy.

**Figure 6:**
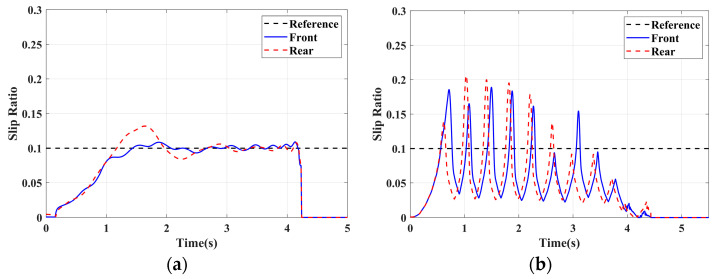
High-adhesion braking test: slip ratio. (**a**) Integrated control (**b**) Rule-based ABS strategy.

**Figure 7:**
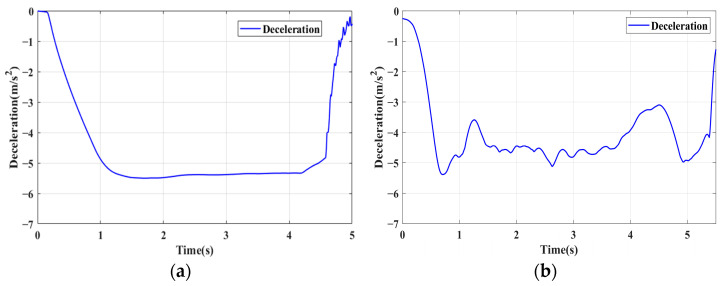
High-adhesion braking test: deceleration. (**a**) Integrated control (**b**) Rule-based ABS strategy.

**Figure 8:**
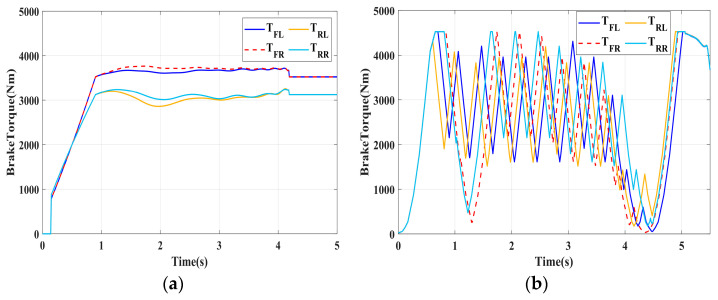
High-adhesion braking test: braking torque. (**a**) Integrated control (**b**) Rule-based ABS strategy.

**Figure 9:**
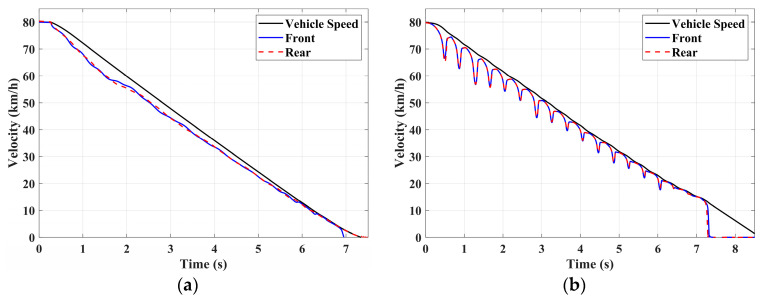
Low-adhesion braking test (initial speed: 80 km/h): vehicle speed and wheel speed. (**a**) Integrated control (**b**) Rule-based ABS strategy.

**Figure 10:**
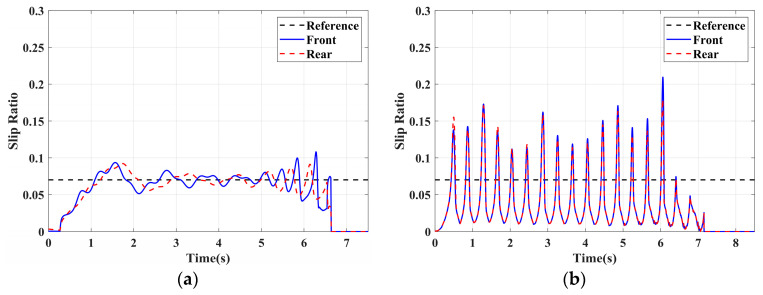
Low-adhesion braking test (initial speed: 80 km/h): slip ratio. (**a**) Integrated control (**b**) Rule-based ABS strategy.

**Figure 11:**
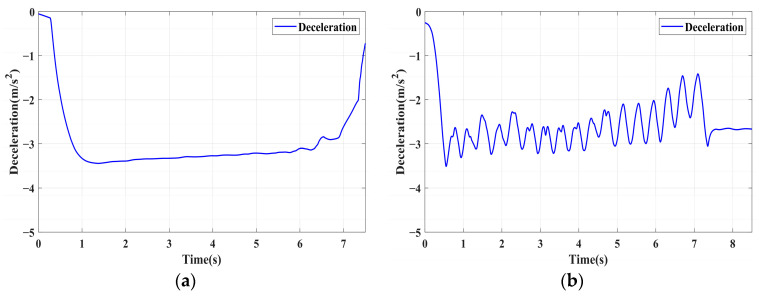
Low-adhesion braking test (initial speed: 80 km/h): deceleration. (**a**) Integrated control (**b**) Rule-based ABS strategy.

**Figure 12:**
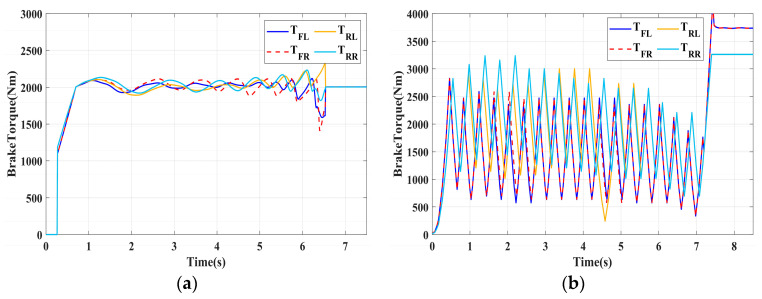
Low-adhesion braking test (initial speed: 80 km/h): braking torque. (**a**) Integrated control (**b**) Rule-based ABS strategy.

**Figure 13:**
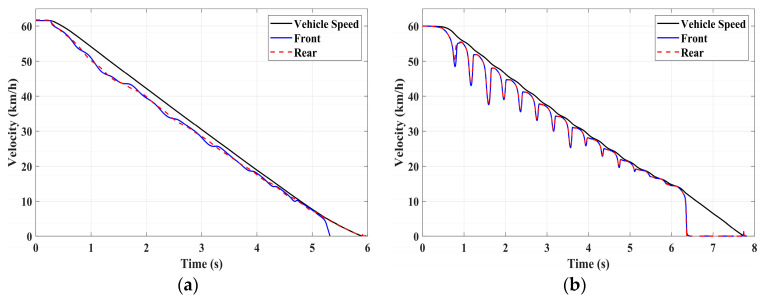
Low-adhesion braking test (initial speed: 60 km/h): vehicle speed and wheel speed. (**a**) Integrated control (**b**) Rule-based ABS strategy.

**Figure 14:**
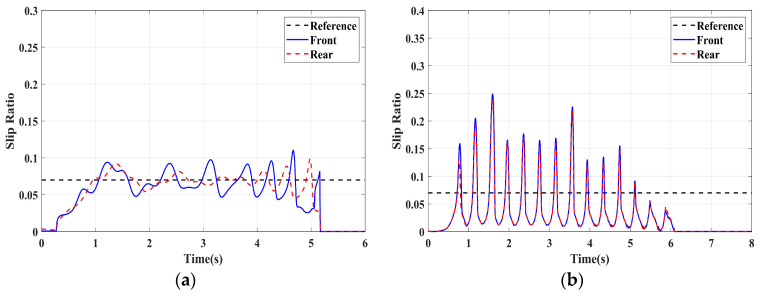
Low-adhesion braking test (initial speed: 60 km/h): slip ratio. (**a**) Integrated control (**b**) Rule-based ABS strategy.

**Figure 15:**
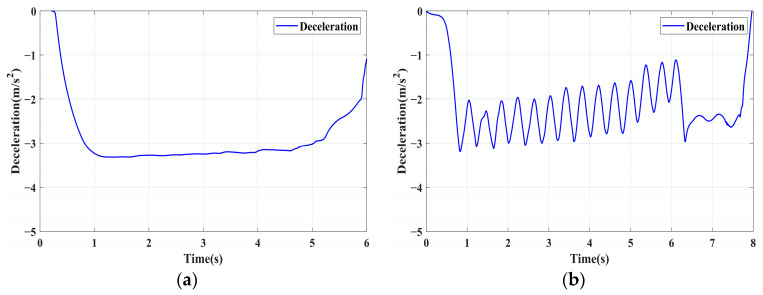
Low-adhesion braking test (initial speed: 60 km/h): deceleration. (**a**) Integrated control (**b**) Rule-based ABS strategy.

**Figure 16:**
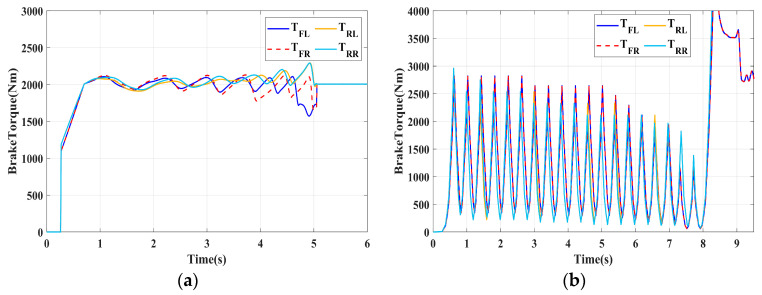
Low-adhesion braking test (initial speed: 60 km/h): braking torque. (**a**) Integrated control (**b**) Rule-based ABS strategy.

In this section, the longitudinal stability of the vehicle with the integrated control strategy was verified through typical braking conditions. Under conditions of good road adhesion, commercial vehicles tend to brake smoothly, and the actual control performance of vehicles with different control strategies does not show significant differences. To thoroughly validate the effectiveness of the proposed control strategy and to allow sufficient optimization space for hard-in-the-loop, rigorous test conditions are selected in this study. Therefore, a road adhesion coefficient of 0.6 is selected to represent a high-adhesion surface, while a coefficient of 0.35 is chosen to represent a low-adhesion surface. The conditions for braking are detailed in Table 4. The brake pedal was pressed from 0 to 100% within 0.1 s to simulate sudden braking. Under the same conditions, the proposed integrated control strategy was compared with the traditional rule-based ABS control strategy. The simulation results for high-adhesion roads are shown in Figure 5, Figure 6, Figure 7 and Figure 8 and Table 5. For low-adhesion roads, the results at an initial speed of 80 km/h are shown in Figure 9, Figure 10, Figure 11 and Figure 12 and Table 6, and at an initial speed of 60 km/h are shown in Figure 13, Figure 14, Figure 15 and Figure 16 and Table 7. The reference slip ratio for high-adhesion roads is 0.1, and for low-adhesion roads it is 0.07. For easier observation of the simulation results, the wheel speed and slip ratio simulation results are selected for the left front wheel and left rear wheel.

The comparison of vehicle speed and wheel speed between the integrated control strategy and rule-based ABS control strategy under high-adhesion road surface testing is shown in Figure 5a,b. Compared with the rule-based ABS strategy, the integrated control strategy, as shown in Figure 5a,b and Table 5, reduces the braking time by 0.81 s and shortens the braking distance by 9 m while ensuring the stability of wheel speed exhibiting better braking performance. Figure 6 shows the comparison of slip ratios between the two strategies. Compared with the traditional rule-based ABS strategy, the proposed integrated control strategy enables better tracking of the ideal slip ratio of the wheels, with the wheel slip ratio always stable near the ideal slip ratio. The wheel slip ratio under the rule-based ABS strategy fluctuates more dramatically, ranging between 0.05 and 0.2. Additionally, because the rule-based ABS strategy is based on thresholds for slip ratio and wheel angular acceleration, it performs poorly in low-speed regions during actual operation. When the vehicle speed is below 15 km/h, the control effect of the rule-based ABS strategy on slip ratio decreases. In contrast, the proposed integrated control strategy effectively addresses this issue. Comparing data in Figure 7 and Table 5, the proposed integrated control strategy achieves an average deceleration of 5.49 m/s^2^ under high adhesion, which is greater than the control group 4.13 m/s^2^. From the comprehensive analysis of the braking forces of each wheel in Figure 8, it can be observed that the braking torque of the integrated control strategy is relatively stable, with actual variation not exceeding 300 Nm. In contrast, the rule-based ABS strategy requires continuous loading and unloading of braking torque, failing to constrain the large fluctuation amplitude of the braking torque effectively.

To fully verify the adaptability of the proposed strategy on various road surfaces, the performance of the integrated control strategy on low-adhesion road surfaces was also tested. The strategy was validated under the test conditions of low-adhesion road surface testing, and the results are shown in Table 6 and Figure 9, Figure 10, Figure 11 and Figure 12.

Table 6 and Figure 9 show the actual braking performance of the two strategies. Under the road conditions of low-adhesion road surface testing, the integrated control strategy reduces the braking time by 1.3 s and the braking distance by 13.7 m, with an average deceleration of 3.4 m/s^2^. These simulations demonstrate that vehicles equipped with the integrated control strategy exhibit better braking performance on low adhesion coefficient roads. Figure 10 and Figure 12 show the slip ratios and braking torques of each wheel for the two strategies. The wheel slip ratio is directly related to the input of braking torque: when the braking torque is released, the slip ratio decreases; when the braking torque increases, the slip ratio rises. The rule-based ABS strategy is implemented based on this logic. According to Figure 10b, Figure 11b and Figure 12b, the actual slip ratio and braking torque fluctuate greatly. Although it can prevent wheel lock-up, the improvement in braking performance is not significant. The integrated control strategy ensures more stable braking torque input, and the slip ratio can closely follow the ideal slip ratio. As shown in Figure 11, under relatively stable braking torques for each wheel, the vehicle deceleration also converges to a constant value.

To thoroughly validate the effectiveness of the strategy, a test was conducted where vehicles braked on a low-adhesion road from an initial speed of 60 km/h with a 70% brake pedal. From Figure 13 and Figure 14 and Table 7, it can be observed that the integrated control strategy still maintains a significant advantage over the traditional rule-based ABS. The braking distance has been reduced by 15 m, and the braking time has decreased by 1.8 s. The variation in slip rate (the difference between the maximum and minimum slip rates) is 0.08 for the integrated control strategy, while it exceeds 0.2 for the traditional rule-based ABS. From Figure 16, it is evident that the braking torque of the integrated control strategy remains consistently around 2000 Nm, while the braking torque of the rule-based ABS fluctuates significantly. This directly leads to differences in deceleration between the two strategies, with the former exhibiting greater stability.

In summary, the integrated control strategy outperforms the rule-based ABS strategy in terms of braking intensity and braking stability on both high-adhesion and low-adhesion road surfaces.

### 4.2. Simulation of Driving Conditions for the Integrated Control Strategy

**Figure 17:**
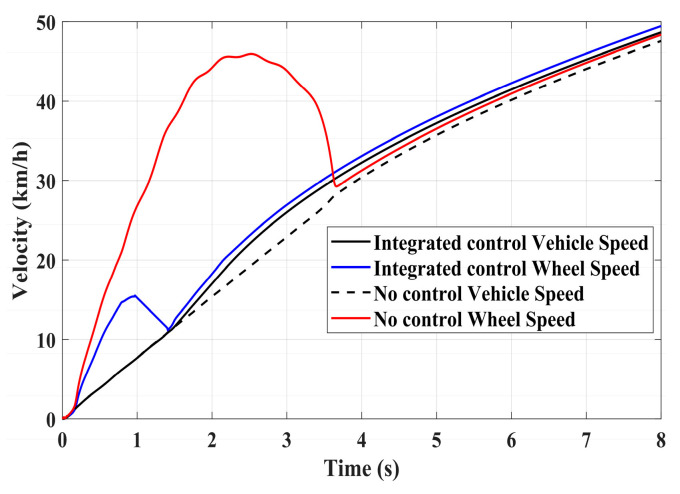
High-adhesion driving test: vehicle speed and wheel speed.

**Figure 18:**
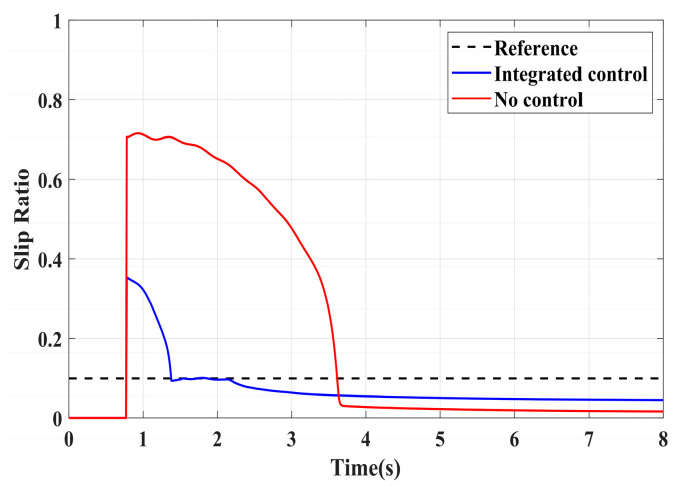
High-adhesion driving test: slip ratio.

**Figure 19:**
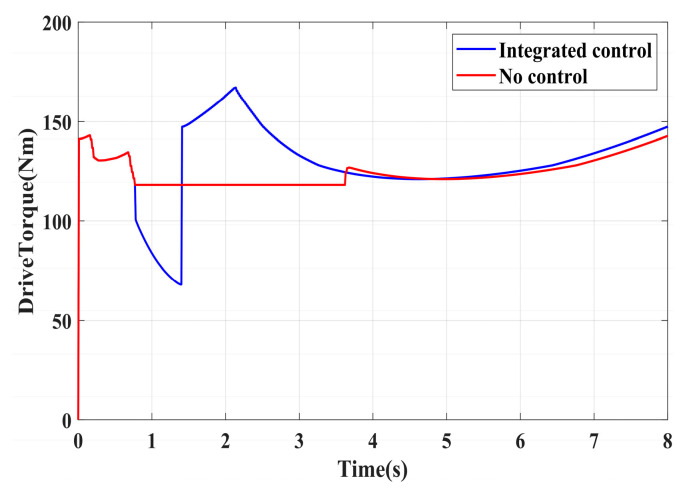
High-adhesion driving test: motor driving torque.

**Figure 20:**
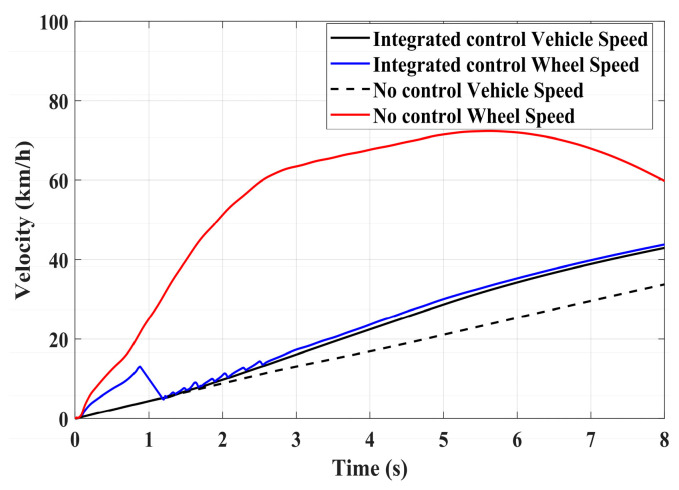
Low-adhesion driving test: vehicle speed and wheel speed.

**Figure 21:**
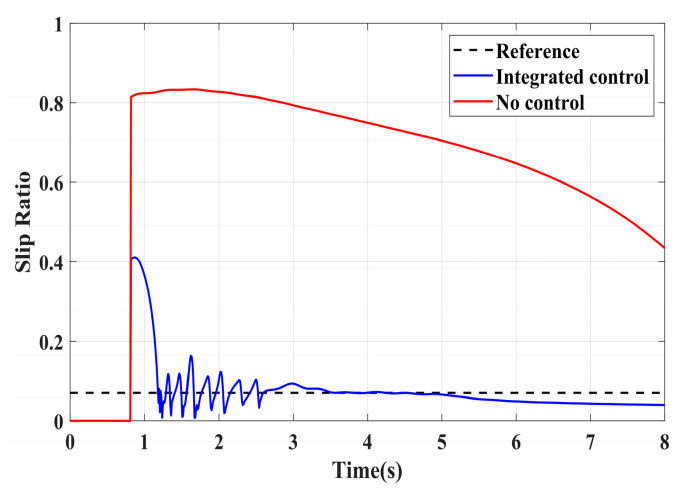
Low-adhesion driving test: slip ratio.

**Figure 22:**
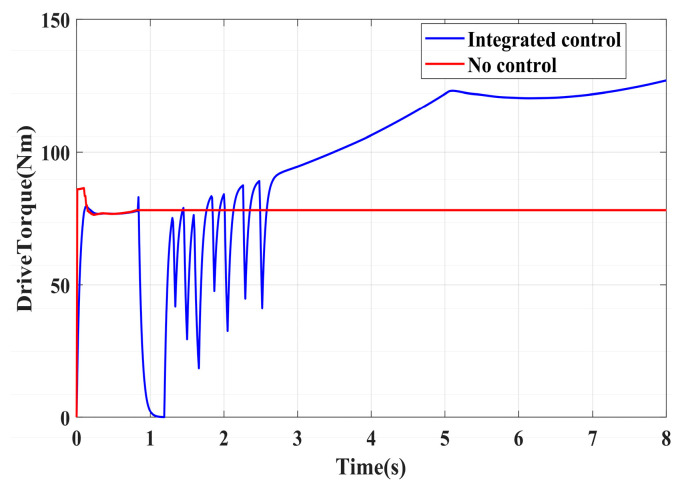
Low-adhesion driving test: motor driving torque.

To validate the effectiveness of the proposed integrated control strategy in terms of longitudinal stability in this paper, we also assess its performance under driving conditions and compare it with vehicles without traction control. The road adhesion coefficient for validating driving conditions is the same as that for braking conditions. A road adhesion coefficient of 0.6 is chosen to represent high-adhesion road surfaces, while a coefficient of 0.35 is selected for low-adhesion road surfaces. Driving conditions are detailed in Table 8. To fully compare the slip conditions under different driving conditions, the accelerator pedal is pressed abruptly in both cases from 0 to 100% within 0.1 s. Simulation results for high-adhesion road surface testing are shown in Figure 17, Figure 18 and Figure 19 and Table 9. Simulation results for low-adhesion road surface testing are shown in Figure 20, Figure 21 and Figure 22 and Table 10.

Figure 17 shows the vehicle speed and driving wheel speed when the vehicle accelerates with a 100% accelerator pedal on a road with a friction coefficient of 0.6. It is evident that vehicles without traction control experience significant wheel slip during acceleration, with the driving wheels remaining in an over-slip state for an extended period, resulting in underutilization of road adhesion. However, the NMPC-based integrated control strategy proposed in this paper can quickly reduce the tendency of over-slip within 1 s. Figure 18 shows the slip ratio of the driving wheels under high-adhesion road surface testing. When the vehicle speed is very low, the influence of road adhesion and vehicle body vibration is significant, which leads to the inaccurate calculation of the slip ratio. Therefore, the slip ratio is not calculated when the vehicle speed is below 5 km/h. Comparing the slip ratios with and without control, the slip ratio of the driving wheels of uncontrolled vehicles reaches a maximum of 0.7 and remains at a high level for 2.9 s. In contrast, under the integrated control strategy, the slip ratio of the driving wheels reaches a maximum of 0.35 and stabilizes near the ideal value within 0.5 s. As the vehicle speed reaches higher speeds, the vehicle gradually stabilizes, and the slip condition of the driving wheels improves significantly. Even with continued acceleration, the slip of the vehicle’s driving wheels is not evident. Therefore, in the latter part of the simulation, the vehicle experiences almost no slip, which is considered ideal. From the motor driving torque in Figure 19, it can be observed that at the beginning of driving, the driving torque of both controlled and uncontrolled vehicles is almost the same. When the slip ratio increases significantly, the integrated control strategy rapidly reduces the motor driving torque to reduce the slip ratio of the driving wheels. Once the slip of the driving wheels stabilizes, the integrated control strategy quickly increases the driving torque again, enhancing the vehicle’s acceleration capability.

Figure 20, Figure 21 and Figure 22 show the simulation results of vehicle speed, slip ratio, and motor torque under a road surface with a coefficient of friction of 0.35, and the vehicle accelerates from an initial speed of 0. From the results of Figure 20 and Figure 21 and Table 10, the uncontrolled vehicle’s driving wheels exhibit a high slip condition during acceleration, with the slip ratio reaching over 0.8, and the vehicle does not return to a normal driving state within 8 s. In contrast, under the integrated control strategy, the slip ratio of the driving wheels stabilizes near the ideal slip ratio within 1.7 s, and the vehicle enters a normal driving state after 5 s, greatly improving vehicle stability during acceleration. Figure 22 shows the performance of the motor driving torque on low-adhesion roads. From the comparison in Figure 22, it is evident that under the integrated control strategy, the motor driving torque changes rapidly during driving wheel slip. The multiple increases and decreases in motor torque significantly improve the slip condition of the driving wheels, which plays a beneficial role in driving on low-adhesion roads.

### 4.3. Simulation of Double-Lane Changes for the Integrated Control Strategy

**Figure 23:**
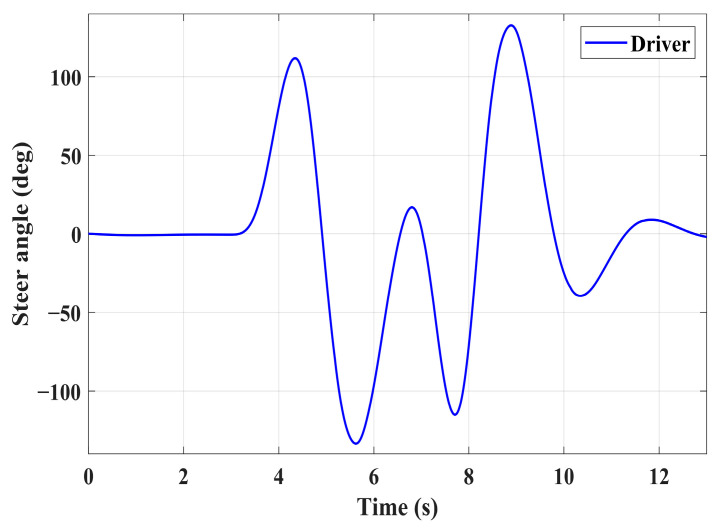
Steering wheel angle under double-lane change conditions high-adhesion and low-adhesion.

**Figure 24:**
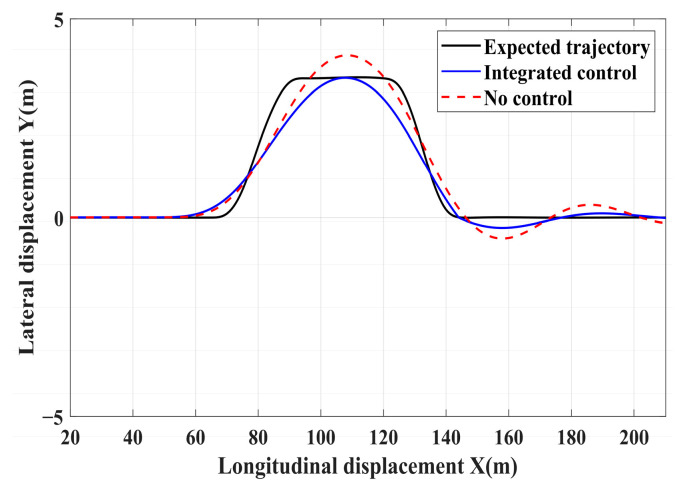
High-adhesion double-lane change test: path-tracking.

**Figure 25:**
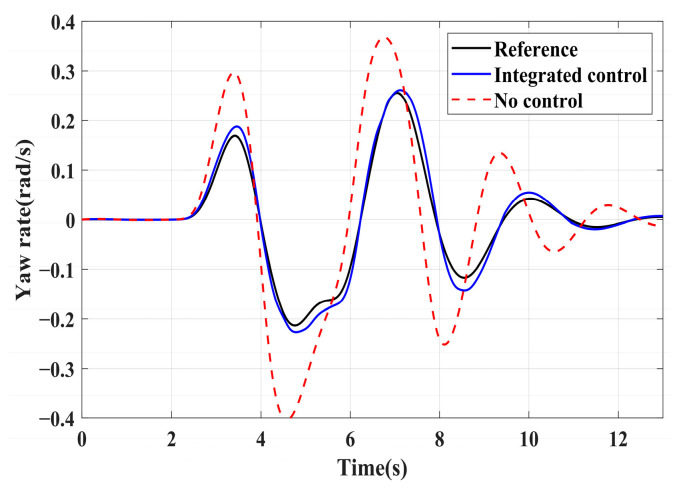
High-adhesion double-lane change test: yaw rate.

**Figure 26:**
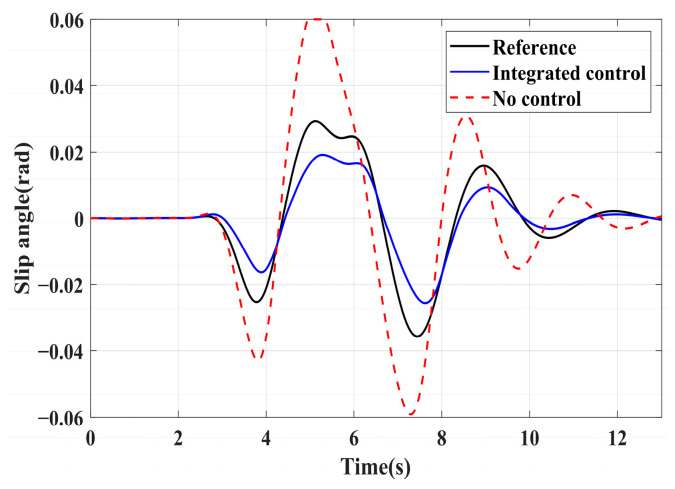
High-adhesion double-lane change test: slip angle.

**Figure 27:**
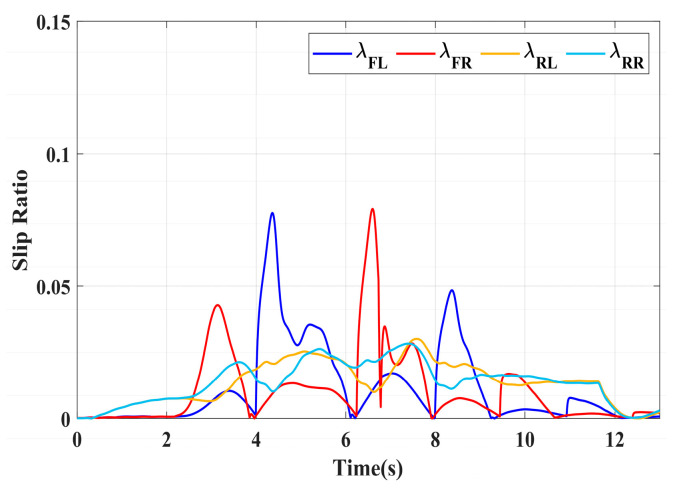
High-adhesion double-lane change test: slip ratio.

**Figure 28:**
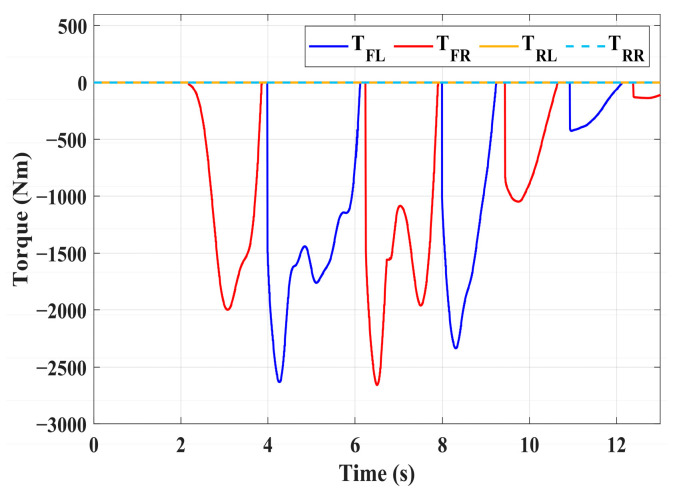
High-adhesion double-lane change test: braking torque.

**Figure 29:**
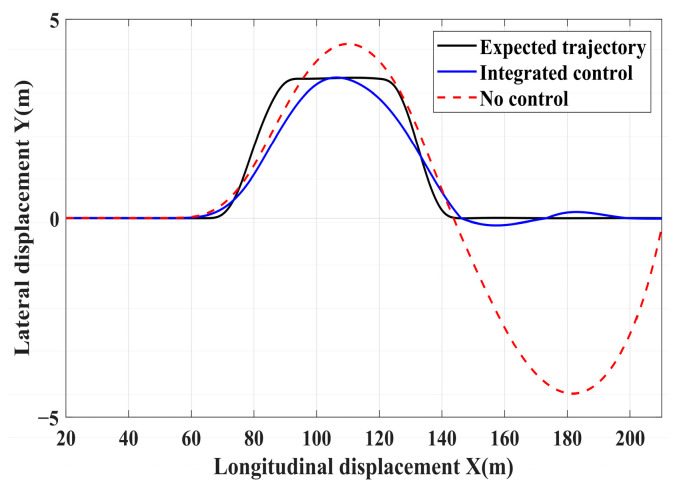
Low-adhesion double-lane change test: path-tracking.

**Figure 30:**
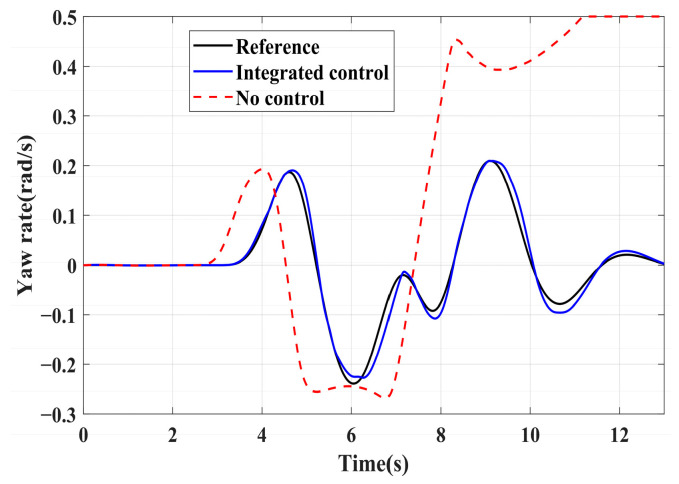
Low-adhesion double-lane change test: yaw rate.

**Figure 31:**
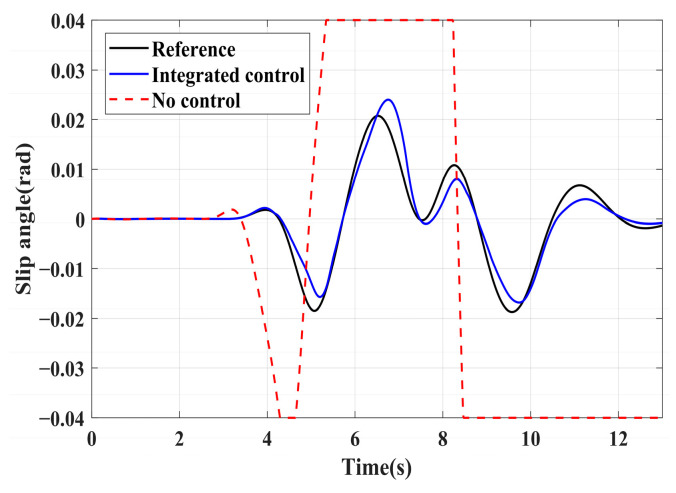
Low-adhesion double-lane change test: slip angle.

**Figure 32:**
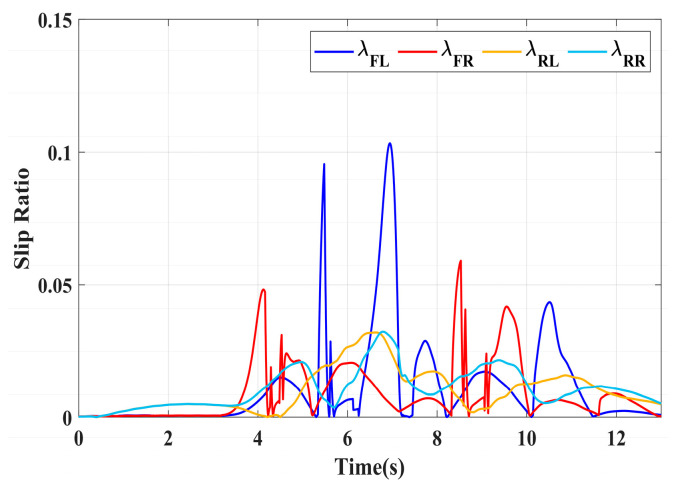
Low-adhesion double-lane change test: slip ratio.

**Figure 33:**
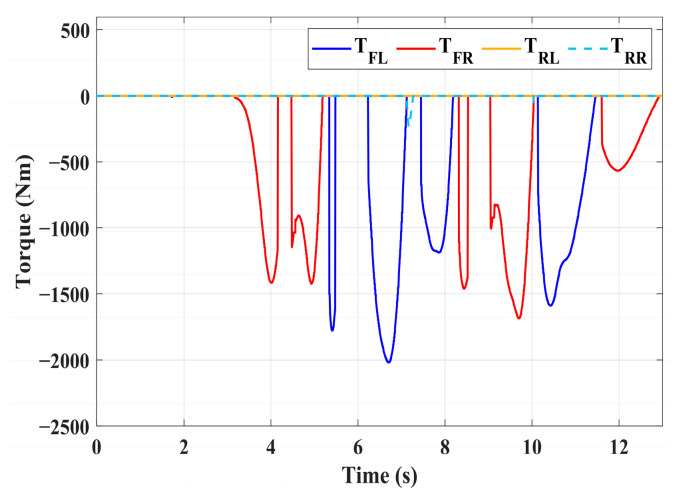
Low-adhesion double-lane change test: braking torque.

In this section, the coordinated lateral and longitudinal stability of the integrated control strategy is verified under double-lane change conditions on both high and low-adhesion roads. A road adhesion coefficient of 0.6 is selected to represent high-adhesion road surfaces, while a coefficient of 0.35 is chosen for low-adhesion road surfaces. The steering wheel angle for the double-lane change condition is shown in Figure 23, and the conditions for the double-lane change are detailed in Table 11. The simulation results for double-lane change high-adhesion road surface testing are shown in Figure 24, Figure 25, Figure 26, Figure 27 and Figure 28, and the simulation results for double-lane change low-adhesion road surface testing are shown in Figure 29, Figure 30, Figure 31, Figure 32 and Figure 33.

For high-adhesion road surface testing, a double-lane change test is conducted with an initial speed of 80 km/h and a road adhesion coefficient of 0.6. The driver steering wheel input is shown in Figure 23. Figure 24 shows the path-tracking simulation results under high-adhesion road surface testing. The results indicate that the vehicle with the integrated control strategy can track the desired path without excessive deviation more successfully than the vehicle without control. Figure 25 and Figure 26 compare the yaw rate and slip angle with and without the integrated control strategy. At 2.3 s, the integrated control strategy begins to intervene, and the lateral stability controller calculates the required braking torque for each wheel to restore stability. The braking torque curves are shown in Figure 28. With the intervention of braking torque on each wheel, the vehicle quickly regains stability, and the yaw rate and slip angle track the ideal values. In this process, the torque output from the lateral stability controller is redistributed in the lower-level longitudinal stability controller to mitigate fluctuations in slip ratio, as depicted in Figure 27. From the slip ratio and braking force curves, it is evident that when there is a risk of excessive slip, the braking torque decreases quickly, thereby reducing the slip ratio. The simulation results convincingly demonstrate the highly effective coordination between lateral and longitudinal control. Compared with the double-lane change test without control, the uncontrolled vehicle starts to destabilize after deviating from the ideal path under the same driver input. The yaw rate deviates from the ideal value, peaking at −0.4 rad/s and 0.37 rad/s at 4.4 s and 6.7 s. The difference between the slip angle and the ideal value reaches a maximum of 0.04 rad.

Low-adhesion road testing conducts a double-lane change with an initial speed of 60 km/h and a road adhesion coefficient of 0.35. The actual driving path following the situation is shown in Figure 29. Due to the low road adhesion coefficient, the uncontrolled vehicle experiences severe deviation when returning to the path, leading to fishtailing and severe instability. The yaw rate and slip angle in Figure 30 and Figure 31 clearly indicate that the uncontrolled vehicle has already lost lateral stability on a low adhesion road, with the yaw rate and slip angle deviating significantly from the ideal values. Vehicles with lateral stability integrated control strategy intervention perform significantly better than uncontrolled vehicles in path following, especially when returning to the original lane without instability, effectively completing the transition. The yaw rate generally follows the ideal value, and the slip angle only slightly exceeds the ideal value at the peak of each steering maneuver, with an error of no more than 0.005 rad. The slip ratio situation shown in Figure 32 indicates that braking torque can be promptly removed when the slip ratio increases, ensuring longitudinal stability. Upon observing the intervention of braking torque for each wheel in Figure 33, the braking torque is generally less than 2000 N, demonstrating the feasibility and reasonableness of introducing braking torque to lateral stability control.

The integrated NMPC control strategy-equipped vehicle demonstrates significant advantages in maintaining yaw stability across both double-lane change conditions. It can ensure stable tracking of the target path on high-adhesion road surfaces, controlling the vehicle yaw rate and slip angle to follow the ideal values. Additionally, it ensures that the vehicle does not experience severe instability on low-adhesion roads, thus maintaining vehicle stability.

## 5. Conclusions

This paper proposes an integrated control strategy that combines the ABS, ASR, and DYC. This strategy mainly addresses the coordination issues among different vehicle controllers. A lateral controller based on NMPC was designed, and on this basis, the control output of the yaw controller is used as the control input for the longitudinal stability controller for secondary judgment. The final output of the longitudinal controller is then transmitted to the actual actuator. This approach not only prevents lateral instability during vehicle motion but also considers the possibility of longitudinal slip. To verify the proposed integrated control strategy, simulation tests were designed for braking, driving, and double-lane change conditions. The test results show that compared with the Rule-based ABS strategy, the proposed strategy improves braking deceleration by an average of 32% and reduces braking distance by 15% on both high and low-adhesion roads, with smaller slip ratio fluctuations during braking. Under driving conditions, the time to enter smooth driving on a high-adhesion road is reduced by an average of 1.5 s, and performance on a low-adhesion road is even better, with the time reduced by nearly 5 s. Under double-lane change conditions on high adhesion surfaces, the vehicle lateral stability parameters are reduced by an average of 50% compared with a vehicle without control. On a low-adhesion road, the vehicle can achieve its predetermined goals well under the proposed integrated strategy.

In summary, the integrated control strategy proposed in this paper successfully completed tests under various conditions, verifying its effectiveness in lateral and longitudinal stability. However, this study has only validated single driving conditions, and further verification is needed for the strategy performance under complex conditions, such as uneven road surfaces. In the future, the existing integrated control strategy will be gradually developed to adapt to more complex conditions.

## Figures and Tables

**Table 1:** Vehicle parameters.

Definition	Unit	Value
Vehicle mass (full)	kg	4495
Vehicle mass (no load)	kg	3200
Front axle load (full)	kg	2000
Front axle load (no load)	kg	1800
Real axle load (full)	kg	2495
Real axle load (no load)	kg	1400
Height of vehicle c.g. (full)	mm	844
Height of vehicle c.g. (no load)	mm	630
Distance between two axles	mm	3300
Wheel type	-	750R16

**Table 2:** Selection of target braking wheel.

Steering Wheel Angle δ	$\mathrm{Reference}\;\mathrm{Yaw}\;\mathrm{Rate}\;{\dot{\varphi }}_{ref}$	$\mathrm{Actual}\;\mathrm{Yaw}\;\mathrm{Rate}\;\dot{\varphi }$	$\mathrm{The}\;\mathrm{Relationship}\;\mathrm{between}\;\dot{\varphi }$ $\;\mathrm{and}\;{\dot{\varphi }}_{ref}$	Steering Characteristic	Braking Wheel
δ>0	${\dot{\varphi }}_{ref}>0$	$\dot{\varphi }>0$	$\dot{\varphi }>{\dot{\varphi }}_{ref}$	Oversteering	Right front wheel
		$\dot{\varphi }>0$	$\dot{\varphi }<{\dot{\varphi }}_{ref}$	Understeering	Left rear wheel
		$\dot{\varphi }<0$	$\dot{\varphi }<0<{\dot{\varphi }}_{ref}$	Understeering	Left rear wheel
δ<0	${\dot{\varphi }}_{ref}<0$	$\dot{\varphi }<0$	$\left|\dot{\varphi }\right|>\left|{\dot{\varphi }}_{ref}\right|$	Oversteering	Left front wheel
		$\dot{\varphi }<0$	$\left|\dot{\varphi }\right|<\left|{\dot{\varphi }}_{ref}\right|$	Understeering	Right rear wheel
		$\dot{\varphi }>0$	${\dot{\varphi }}_{ref}<0<\dot{\varphi }$	Understeering	Right rear wheel

**Table 3:** Road Condition Parameters.

Road Conditions	$c_{1}$	$c_{2}$	$c_{3}$
dry asphalt	1.2801	23.99	0.52
wet asphalt	0.857	33.822	0.52
dry concrete	1.1973	25.168	0.5373
cobble wet	0.4004	33.708	0.1204
cobble dry	1.3713	6.4565	0.6691
snow	0.1946	94.129	0.0646
ice	0.05	306.39	0

**Table 4:** Braking condition settings.

Condition	Road Adhesion	Initial Speed (km/h)	Brake Pedal (%)
High-adhesion test	*µ* = 0.6	80	100
Low-adhesion test	*µ* = 0.35	80	100
Low-adhesion test	*µ* = 0.35	60	70

**Table 5:** Simulation results of high-adhesion braking.

Definition	Unit	Value (Integrated Control)	Value (Rule-Based)
Braking time	s	4.57	5.38
Average deceleration	m/s^2^	5.49	4.13
Braking distance	m	50.78	59.78
Maximum slip rate	-	0.13	0.20
Minimum slip rate	-	0.08	0.025

**Table 6:** Simulation results of low-adhesion braking (initial speed: 80 km/h).

Definition	Unit	Value (Integrated Control)	Value (Rule-Based)
Braking time	s	7.33	8.63
Average deceleration	m/s^2^	3.40	2.57
Braking distance	m	81.44	95.14
Maximum slip rate	-	0.11	0.21
Minimum slip rate	-	0.04	0.015

**Table 7:** Simulation results of low-adhesion braking (initial speed: 60 km/h).

Definition	Unit	Value (Integrated Control)	Value (Rule-Based)
Braking time	s	6.0	7.8
Average deceleration	m/s^2^	3.2	2.34
Braking distance	m	50	65
Maximum slip rate	-	0.12	0.25
Minimum slip rate	-	0.04	0.015

**Table 8:** Driving condition settings.

Condition	Road Adhesion	Initial Speed (km/h)	Accelerator Pedal (%)
High-adhesion test	*µ* = 0.6	0	100
Low-adhesion test	*µ* = 0.35	0	100

**Table 9:** Simulation results of high-adhesion driving.

Definition	Unit	Value (Integrated Control)	Value (Rule-Based)
Acceleration time 0–30 km/h	s	3.50	3.98
Acceleration slip time	s	0.5	2.90
Maximum slip rate	-	0.35	0.70

**Table 10:** Simulation results of low-adhesion driving.

Definition	Unit	Value (Integrated Control)	Value (Rule-Based)
Acceleration time 0–30 km/h	s	5.0	7.0
Acceleration slip time	s	1.7	9.0
Maximum slip rate	-	0.40	0.82

**Table 11:** Double-lane change condition settings.

Condition	Road Adhesion	Initial Velocity (km/h)
High-adhesion test	*µ* = 0.6	80
Low-adhesion test	*µ* = 0.35	60

## Data Availability

Data are contained within the article.
